# Japanese subpopulation analysis of MONARCH 2: phase 3 study of abemaciclib plus fulvestrant for treatment of hormone receptor-positive, human epidermal growth factor receptor 2-negative breast cancer that progressed on endocrine therapy

**DOI:** 10.1007/s12282-021-01239-8

**Published:** 2021-04-01

**Authors:** Kenichi Inoue, Norikazu Masuda, Hiroji Iwata, Masato Takahashi, Yoshinori Ito, Yasuo Miyoshi, Takahiro Nakayama, Hirofumi Mukai, Jan-Stefan van der Walt, Joji Mori, Sachi Sakaguchi, Tsutomu Kawaguchi, Yoshinori Tanizawa, Antonio Llombart-Cussac, George W. Sledge, Masakazu Toi

**Affiliations:** 1 Saitama Cancer Center, Saitama, Japan; 2 National Hospital Organization, Osaka National Hospital, Osaka, Japan; 3 Department of Breast Oncology, Aichi Cancer Center Hospital, Nagoya, Japan; 4 National Hospital Organization Hokkaido Cancer Center, Sapporo, Japan; 5 The Cancer Institute Hospital, Tokyo, Japan; 6 Hyogo College of Medicine, Hyogo, Japan; 7 Osaka International Cancer Institute, Osaka, Japan; 8 National Cancer Center Hospital East, Kashiwa, Japan; 9 Eli Lilly and Company, Surrey, UK; 10 Eli Lilly Japan K.K., Kobe, Japan; 11 Hospital Arnau de Vilanova, Valencia, Spain; 12 SOLTI Breast Cancer Research Group, Barcelona, Spain; 13 Stanford University School of Medicine, Stanford, CA USA; 14 Breast Cancer Unit, Breast Surgery, Graduate School of Medicine, Kyoto University Hospital, Kyoto University, 54 Shogoin-Kawaracho, Sakyo-ku, Kyoto, 606-8507 Japan

**Keywords:** Abemaciclib, Breast cancer, Cyclin-dependent kinase 4 and 6 inhibitor

## Abstract

**Background:**

This was a Japanese subpopulation analysis of MONARCH 2, a double-blind, randomized, placebo-controlled, phase 3 study of abemaciclib plus fulvestrant in patients with hormone receptor-positive, human epidermal growth factor receptor 2-negative advanced breast cancer (ABC).

**Methods:**

Eligible women had progressed on (neo)adjuvant endocrine therapy (ET), ≤ 12 months from end of adjuvant ET, or on first-line ET for ABC, and had not received chemotherapy for ABC. Patients were randomized 2:1 to receive abemaciclib or placebo plus fulvestrant. The primary endpoint was progression-free survival (PFS). Secondary endpoints included overall survival (OS), pharmacokinetics (PK), health-related quality of life (HRQoL), and safety.

**Results:**

In Japan, 95 patients were randomized (abemaciclib, *n* = 64; placebo, *n* = 31). At final PFS analysis (February 14, 2017), median PFS was 21.2 and 14.3 months, respectively, in the abemaciclib and placebo groups (hazard ratio: 0.672; 95% confidence interval: 0.380–1.189). Abemaciclib had a higher objective response rate (37.5%) than placebo (12.9%). PK and safety profiles for Japanese patients were consistent with those of the overall population, without clinically meaningful differences across most HRQoL dimensions evaluated. The most frequent adverse events in the abemaciclib versus placebo groups were diarrhea (95.2 versus 25.8%), neutropenia (79.4 versus 0%), and leukopenia (66.7 versus 0%). At a second data cutoff (June 20, 2019), median OS was not reached with abemaciclib and 47.3 months with placebo (hazard ratio: 0.755; 95% confidence interval: 0.390–1.463).

**Conclusions:**

Results of the Japanese subpopulation were consistent with the improved clinical outcomes and manageable safety profile observed in the overall population.

**Clinical trial registration:**

NCT02107703; U.S. National Library of Medicine: https://clinicaltrials.gov/ct2/show/NCT02107703.

**Supplementary Information:**

The online version contains supplementary material available at 10.1007/s12282-021-01239-8.

## Introduction

Breast cancer is the second leading cause of cancer mortality in women globally [1]. Women diagnosed with hormone receptor-positive (HR+), human epidermal growth factor receptor 2-negative (HER2−) breast cancer are typically treated with endocrine therapy (ET), but intrinsic and acquired ET resistance are common issues in patients with advanced or metastatic breast cancer [2, 3].The cyclin D pathway is an important target for overcoming mechanisms of ET resistance [4]. Targeting this pathway with cyclin-dependent kinase 4 and 6 (CDK4 and CDK6) inhibitors in combination with ET results in significant improvement in clinical outcomes over ET alone [5–15], with CDK4 and CDK6 inhibitor/ET combination therapy emerging as the new standard of care in the treatment of HR+ , HER2− advanced breast cancer (ABC) [16].

Abemaciclib is a selective small molecule inhibitor of CDK4 and CDK6 orally administered on a continuous twice-daily dosing regimen [17–19]. In preclinical cancer models, continuous inhibition of CDK4 and CDK6 by abemaciclib led to cell cycle arrest and death of cancer cells [18, 20]. Within the abemaciclib clinical development program, MONARCH 2 was a randomized, double-blind, global, phase 3 study of abemaciclib in combination with fulvestrant in women with HR+ , HER2− ABC whose disease had progressed while receiving prior ET. In the intent-to-treat (ITT) population, abemaciclib plus fulvestrant significantly extended both progression-free survival (PFS; median: 16.4 versus 9.3 months, hazard ratio [HR]: 0.553, 95% confidence interval [CI]: 0.449–0.681, *p* < 0.001) and overall survival (OS; median: 46.7 versus 37.3 months, HR: 0.757; 95% CI: 0.606–0.945; *p* = 0.01) compared with placebo plus fulvestrant [12, 13].

Based on the findings of the global clinical development program, abemaciclib was approved for use in Japan in September 2018 in combination with ET for the treatment of HR+ , HER2− ABC. However, ethnicity and country-specific differences in clinical practice can influence response to breast cancer treatment [21, 22], and potential interethnic differences in response to abemaciclib have not been extensively studied. The objective of the current analysis was to assess efficacy and safety outcomes in Japanese breast cancer patients within the MONARCH 2 population. Here, we report PFS, safety, patient-reported health-related quality of life (HRQoL), and pharmacokinetic (PK) outcomes of the Japanese subpopulation of MONARCH 2 at the time of the final PFS analysis. In addition, we report OS, time to chemotherapy (TTC), chemotherapy-free survival (CFS), and updated PFS and safety of the Japanese subpopulation at a second data cutoff date, 27 months following the final PFS analysis.

## Patients and methods

### Study design and patients

This analysis was conducted on patients enrolled at study sites in Japan for the global MONARCH 2 study (NCT02107703), a randomized, double-blind, placebo-controlled study of abemaciclib plus fulvestrant in women with ABC (Online Resource 1). Study design and methods for MONARCH 2 have previously been published [12, 13, 23]. Patients were required to have HR+ , HER2− inoperable locally advanced or metastatic breast cancer that progressed on neoadjuvant/adjuvant ET, ≤ 12 months from end of adjuvant ET, or on first-line ET for ABC and who had not received chemotherapy for advanced disease. Patients were excluded if they had prior treatment with fulvestrant, everolimus, or CDK4 and CDK6 inhibitors, or had visceral crisis or evidence/history of central nervous system metastasis.

The study was conducted in accordance with the 1964 Declaration of Helsinki and its later amendments and the relevant laws and regulations in Japan. The protocol was reviewed by ethical and institutional review boards at the participating institutions. Informed consent was obtained from all individual participants in the study.

### Treatments and procedures

Patients were randomized 2:1 to receive abemaciclib plus fulvestrant or placebo plus fulvestrant, stratified by metastatic site and resistance to prior ET (primary or secondary, as defined in European Society for Medical Oncology guidelines [24, 25]). Fulvestrant (500 mg, per label) was administered by intramuscular injection on Days 1 and 15 of the first cycle and on Day 1 of subsequent cycles (every 28 days). Abemaciclib (200 mg; reduced to 150 mg following a protocol amendment [13]) or placebo was administered orally twice daily. Permitted dose adjustments in MONARCH 2 were previously described [13]. Treatment continued until progressive disease (PD), death, or patient withdrawal. Crossover between treatment groups was not permitted. Response was determined by investigators for all patients whose disease was evaluable using Response Evaluation Criteria In Solid Tumors (RECIST) version (v) 1.1 [26].

### Outcomes

Efficacy analyses included all patients in the ITT Japanese subpopulation, regardless of starting dose for abemaciclib. The primary efficacy endpoint was the comparison of PFS between treatment groups. Secondary efficacy outcomes included OS, objective response rate (ORR; the proportion of patients with a best response of complete [CR] or partial response [PR]), disease control rate (DCR; CR+ PR+ stable disease), and clinical benefit rate (CBR; CR+ PR+ stable disease ≥ 6 months).

Additional secondary endpoints included safety, PK, and HRQoL, including global health status, functioning, and symptoms. Safety was evaluated in all patients who received at least one dose of study treatment, with treatment-emergent adverse events (TEAEs) summarized using Medical Dictionary for Regulatory Activities (MedDRA) v.19.1 terminology and graded based on the National Cancer Institute Common Terminology Criteria for Adverse Events (CTCAE) v.4.0. PK analyses were conducted on patients in the safety population who had plasma samples collected, which were obtained at prescheduled times on Days 1 (2–4 h postdose) and 15 (4–7.0 h postdose) of Cycle 1 and on Day 1 of Cycle 2 (predose and 3.0 h postdose) and Cycle 3 (predose). The concentration of abemaciclib in plasma was measured using validated liquid chromatography/tandem mass spectrometry assays.

HRQoL analyses included all patients who completed baseline assessment plus at least one post-baseline assessment, as described [23]. Data were collected using the European Organization for Research and Treatment of Cancer Quality of Life Questionnaire-Core 30 (EORTC QLQ-C30) to assess cancer-related QoL [27] and the EORTC QLQ-Breast Cancer module (EORTC QLQ-BR23) to assess breast cancer-specific QoL [28].

Exploratory endpoints included TTC, CFS, and time to sustained deterioration (TTSD) on the EORTC-QLQ-C30 and QLQ-BR23.

### Statistical analyses

Analyses were conducted at two database locks. PFS, safety, HRQoL, TTSD on HRQoL measures, and PK outcomes are reported at the data cutoff date of February 14, 2017. OS and exploratory endpoints (TTC, CFS) are reported at a second data cutoff date of June 20, 2019, at which time updates on PFS and safety are also provided. Statistical methods for MONARCH 2 have been previously described [12, 13, 23]. For this subpopulation analysis, *p* values for comparisons between treatments are not reported due to the limited sample size. Interim and final PFS and OS analyses were preplanned (Online Resource 1) [12, 13]. PFS, OS, CFS, and TTC were estimated using the Kaplan–Meier method [29], and a Cox proportional hazard model was used to estimate the HRs and corresponding 95% CIs. Normal approximation was used to estimate 95% CIs for ORR, DCR, CBR, and the difference in 36-month PFS rates between the treatment groups.

For EORTC-QLQ-C30 and QLQ-BR23, a score ranging from 0 to 100 was calculated for each scale, with a higher score representing more severe symptoms for symptom scales and better health condition for global health status and functioning scales. Change from baseline over the entire treatment course was assessed using mixed effects-repeated measures models including all data and cycles for which at least 25% of patients completed questionnaires in both study groups. Post hoc analyses investigated TTSD using Cox proportional hazard models. A minimally important difference (MID) of ≥ 10-points [30], considered herein as a clinically meaningful difference, was utilized for TTSD and change from baseline analyses. TTSD for each scale was defined as the time from randomization to the time at which a ≥ 10-point worsening compared with a patient’s baseline score was observed, followed by all subsequent scores meeting MID criteria compared with baseline [31].

A mechanistic population PK model was used to characterize the PK of abemaciclib in the MONARCH 2 population [32]. The resulting model parameter estimates were used to simulate individual patient exposure metrics, including area under the concentration-versus-time curve during one dosing interval at steady state (AUC_τ,ss_), maximum concentration at steady-state (C_max,ss_), and minimum/trough concentration at steady state (C_min,ss_), and summarized to compare the study PK population with the Japanese PK subpopulation.

## Results

### Patients

The MONARCH 2 study enrolled 669 patients between August 7, 2014, and December 29, 2015, 446 and 223 of whom were allocated to receive abemaciclib plus fulvestrant or placebo plus fulvestrant, respectively. Of these, 95 patients were enrolled in Japan (abemaciclib, *n* = 64; placebo, *n* = 31; Online Resource 2). In the abemaciclib group, 20 patients in the Japanese subpopulation (31.3%) initially received a 200 mg dose before a mandatory dose reduction to 150 mg (in comparison with 27.4% of the overall population [13]). At the time of data cutoff for the final PFS analysis, 30 (46.9%) and 8 (25.8%) patients in the abemaciclib and placebo groups of the Japanese subpopulation, respectively, were still on-treatment. The reason for discontinuation of study drug was most frequently PD (abemaciclib: *n* = 27, 42.2%; placebo: *n* = 23, 74.2%).

Table 1 summarizes demographic and baseline clinical characteristics by treatment group. Across treatment groups in the Japanese subpopulation, the majority of patients (60.0%) were post-menopausal with a median age of 58.0 (min–max, 32.0–81.0) years. Approximately half (50.5%) had visceral disease, and the majority had secondary ET resistance (≥ 71%), Eastern Cooperative Oncology Group performance status (ECOG PS) scores of 0 (≥ 84%), and progesterone receptor-positive tumors (> 82%). Although the Japanese subpopulation was generally comparable to the overall MONARCH 2 population for most baseline characteristics, a lower proportion of patients in the Japanese subpopulation were post-menopausal (60.0%) compared with the overall population (82.4%; Table 1). In addition, the Japanese subpopulation had a lower proportion of patients (9.5%) with an ECOG PS score of 1 compared with the overall population (39.3%). Among abemaciclib-treated patients, a lower proportion had primary ET resistance (18.8%) and prior adjuvant chemotherapy (31.3%) in the Japanese ITT population compared with the overall ITT population (24.9 and 46.9%, respectively), resulting in an imbalance between treatment arms only within the Japanese subpopulation (Table 1).

**Table 1:** Baseline demographics and clinical characteristics

Characteristic		Japanese ITT population (*N* = 95)		Overall ITT population (*N* = 669)	
		Abemaciclib + fulvestrant (*n* = 64)	Placebo + fulvestrant (*n* = 31)	Abemaciclib + fulvestrant (*n* = 446)	Placebo + fulvestrant (*n* = 223)
Age, years	Median (range)	56.5 (32–76)	58.0 (32–81)	59.0 (32–91)	62.0 (32–87)
Metastatic site, *n* (%)	Visceral Bone only Other	35 (54.7) 11 (17.2) 18 (28.1)	13 (41.9) 8 (25.8) 10 (32.3)	245 (54.9) 123 (27.6) 75 (16.8)	128 (57.4) 57 (25.6) 38 (17.0)
Menopausal status, *n* (%)	Pre/peri- Post	26 (40.6) 38 (59.4)	12 (38.7) 19 (61.3)	72 (16.1) 371 (83.2)	42 (18.8) 180 (80.7)
Progression while receiving neoadjuvant/adjuvant ET, *n* (%)	Yes	25 (39.1)	17 (54.8)	197 (44.2)	103 (46.2)
ET sensitivity	Primary resistance	12 (18.8)	9 (29.0)	111 (24.9)	58 (26.0)
	Secondary resistance	52 (81.3)	22 (71.0)	326 (73.1)	163 (73.1)
Prior chemotherapy	Neoadjuvant Adjuvant	10 (15.6) 20 (31.3)	9 (29.0) 14 (45.2)	75 (16.8) 209 (46.9)	40 (17.9) 103 (46.2)
ECOG PS, *n* (%)	0 1	60 (93.8) 4 (6.3)	26 (83.9) 5 (16.1)	264 (59.2) 176 (39.5)	136 (61.0) 87 (39.0)
PgR, *n* (%)	Positive	53 (82.8)	27 (87.1)	339 (76.0)	171 (76.7)

*ECOG PS* Eastern Cooperative Oncology Group Performance Status, *ET* endocrine therapy, *ITT* intent-to-treat, *N* number of patients in analysis population, *n* number of patients in category or group, *PgR* progesterone receptor

## Efficacy

**Fig. 1:**
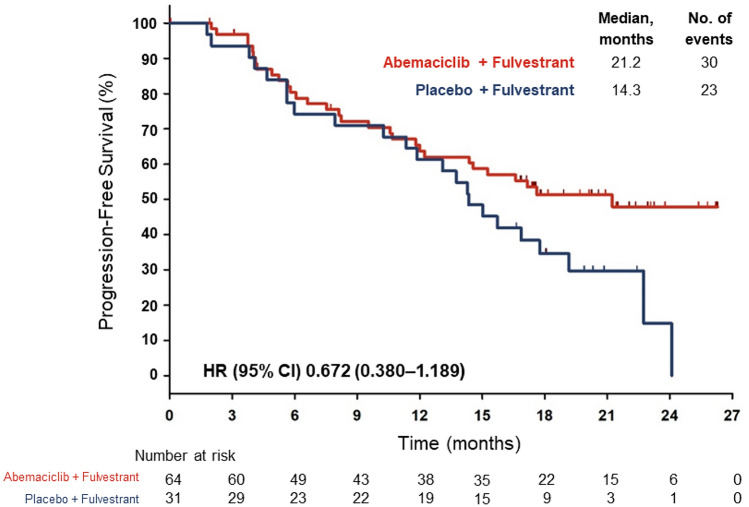
Progression-free survival. PFS analysis at the February 14, 2017 data cutoff date for the MONARCH 2 Japanese subpopulation. PFS was defined as the time from the date of randomization until the date of radiographic documentation of progression, based on investigator assessment, or the date of death, whichever was earlier. The curves and medians (95% CI) were estimated using the Kaplan–Meier method. *CI* confidence interval, *HR* hazard ratio, *No.* number, *PFS* progression-free survival

At the final PFS analysis (data cutoff date: February 14, 2017; median follow-up time 19.5 months), 53 PFS events (abemaciclib: *n* = 30, 46.9%; placebo: *n* = 23, 74.2%) were observed in the Japanese subpopulation. The abemaciclib group had a median PFS of 21.2 months compared with 14.3 months in the placebo group (HR: 0.672; 95% CI: 0.380–1.189; Fig. 1). In the Japan subpopulation, compared with the control group, the abemaciclib group had a higher proportion of patients with a best response of CR or PR (ORR: abemaciclib, 37.5%; placebo, 12.9%; Table 2). The proportion of patients with PD was higher in the placebo group (6.5%) compared with the abemaciclib group (3.1%).

**Table 2:** Summary of best overall response in the Japanese subpopulation of MONARCH 2

Best overall response^a^	Abemaciclib + fulvestrant (*N* = 64)		Placebo + fulvestrant (*N* = 31)	
	*n* (%)	95% CI^b^	*n* (%)	95% CI^b^
Complete response (CR)	2 (3.1)	− 1.1, 7.4	0 (0.0)	NA
Partial response (PR)	22 (34.4)	22.7, 46.0	4 (12.9)	1.1, 24.7
Stable disease (SD)	36 (56.3)	44.1, 68.4	25 (80.6)	66.7, 94.6
SD persistent for ≥ 6 months	26 (40.6)	28.6, 52.7	19 (61.3)	44.1, 78.4
Progressive disease (PD)	2 (3.1)	− 1.1, 7.4	2 (6.5)	− 2.2, 15.1
Objective PD	2 (3.1)	− 1.1, 7.4	2 (6.5)	− 2.2, 15.1
Not evaluable	2 (3.1)	− 1.1, 7.4	0 (0.0)	NA
Objective response rate (CR + PR)	24 (37.5)	25.6, 49.4	4 (12.9)	1.1, 24.7
Disease control rate (CR + PR + SD)	60 (93.8)	87.8, 99.7	29 (93.5)	84.9, 102.2
Clinical benefit rate (CR + PR + SD ≥ 6 months)	50 (78.1)	68.0, 88.3	23 (74.2)	58.8, 89.6

Data cutoff date: February 14, 2017

*CI* confidence interval, *N* number of patients in population, *n* number of patients, *NA* not applicable, *RECIST* Response Evaluation Criteria in Solid Tumors

^a^Response was determined by investigators for all patients whose disease was evaluable using RECIST version 1.1

^b^CIs were based on normal approximation

### Treatment exposure and pharmacokinetics

Dose adjustments and exposure for the Japanese subpopulation of MONARCH 2 are summarized in Online Resource 3. Median duration of abemaciclib/placebo was 64.7 and 65.0 weeks in the abemaciclib and placebo groups, respectively. Median duration of fulvestrant was 75.0 and 65.0 weeks in the abemaciclib and placebo groups, respectively. The median dose intensity for abemaciclib was 231.2 mg/day, and median relative dose intensity was 69.7%. The dose reduction rate and dose omission rate for abemaciclib due to an adverse event (AE) was 54.0 and 82.5%, respectively (placebo, 3.2 and 19.4%, respectively; Online Resource 3). For comparison, in the overall ITT population, the median dose intensity for abemaciclib was 273.1 mg/day and median relative dose intensity was 79.8%, with dose reduction and omission rates due to AEs 42.9 and 51.9%, respectively (placebo, 1.3 and 11.7%, respectively).

**Fig. 2:**
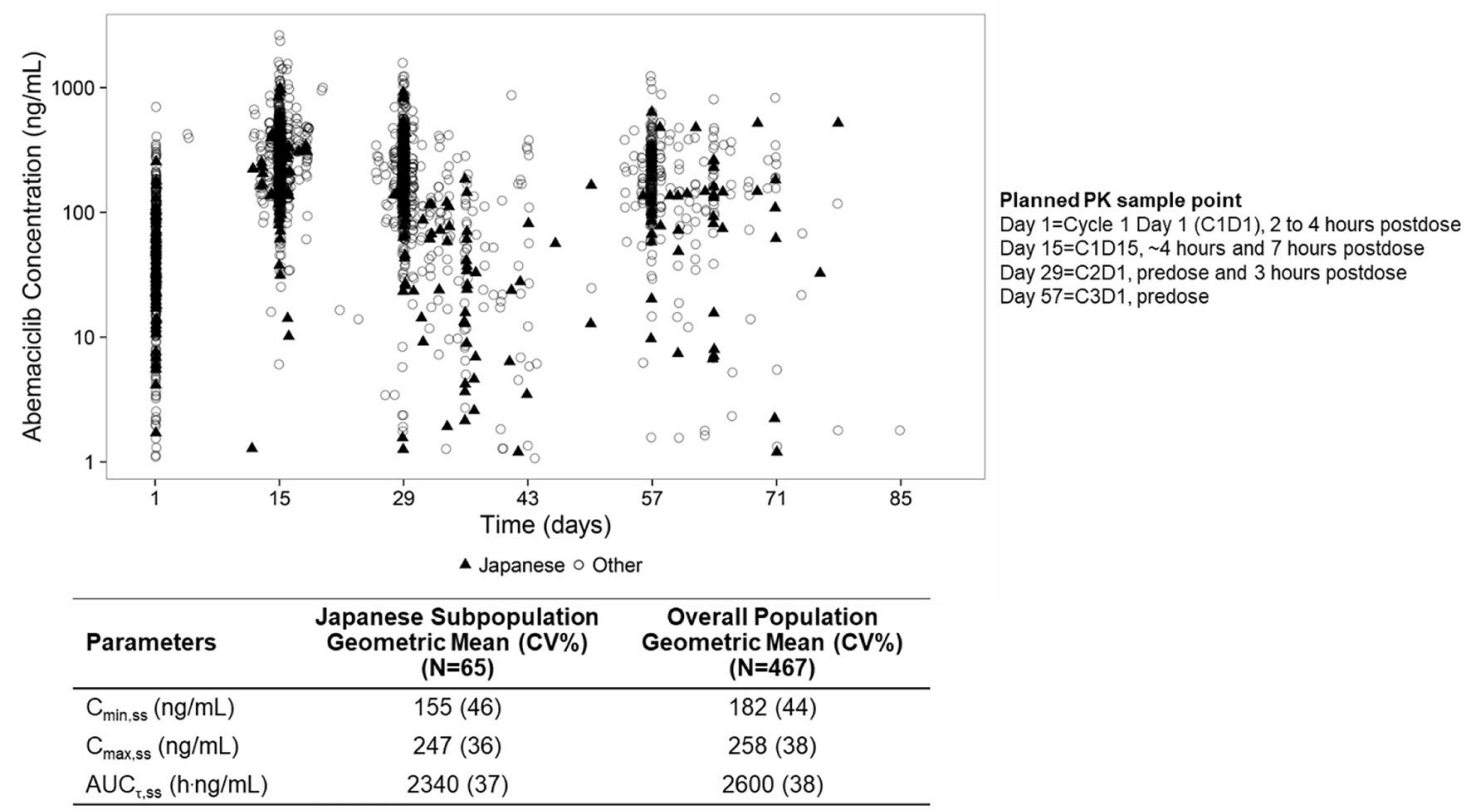
Pharmacokinetic analysis of abemaciclib in patients receiving abemaciclib plus fulvestrant^a^. Blood samples for assessment of abemaciclib concentration in plasma were obtained at the indicated prescheduled times on Days 1 and 15 of Cycle 1 and on Day 1 of Cycles 2 and 3 and measured with a validated assay. Plasma concentrations of abemaciclib for individual patients over the course of the analysis are shown in the top graph, with geometric mean trough and peak concentrations (CV%) for the Japanese subpopulation and MONARCH 2 study population summarized in the table. ^a^The PK analyses are for patients receiving the 200-mg dose (pre-amendment dose) and patients receiving the 150-mg dose (post-amendment dose) combined. PK analyses of abemaciclib were conducted on patients who had received at least 1 dose of abemaciclib and had PK samples collected, and included ET-naïve patients who were excluded from the ITT population*.* *AUC*_*τ,ss*_ area under the concentration versus time curve during one dosing interval at steady state, *C*_*max,ss*_ maximum concentration at steady-state, *C*_*min,ss*_ minimum/trough concentration at steady state, *CV* coefficient of variation, *ET* endocrine therapy, *ITT* intent-to-treat, *PK* pharmacokinetics

Plasma concentrations of abemaciclib for individual patients over the course of the analysis are shown in Fig. 2. Abemaciclib PK steady-state exposure metrics (AUC_τ,ss_, C_max,ss_, C_min,ss_) in the Japanese PK subpopulation were similar to the overall MONARCH 2 PK analysis population, with comparable inter-individual variability.

### Safety

At the primary endpoint analysis (February 14, 2017), all patients in both treatment groups in the Japanese subpopulation reported at least 1 TEAE, with a higher proportion of patients in the abemaciclib group reporting grade 3 (68.3%) or grade 4 (6.3%) TEAEs compared with the placebo group (grade 3, 22.6%; grade 4, 0%). No grade 5 TEAEs were reported in the Japanese subpopulation.

The most common TEAE in both treatment groups was diarrhea, which was reported at a higher frequency in the abemaciclib group (any grade, 95.2%; grade ≥ 3, 14.3%) compared with the placebo group (any grade, 25.8%; grade ≥ 3, 3.2%; Table 3). This is similar to the frequency for TEAEs of diarrhea reported in the global population (any grade, abemaciclib: 86.4%; placebo: 24.7% [13]). The abemaciclib group also reported a higher frequency of hematologic events, including neutropenia (abemaciclib: any grade, 79.4%; grade ≥ 3, 44.4%; placebo: any grade, 0%); leukopenia (abemaciclib: any grade, 66.7%; grade ≥ 3, 20.6%; placebo: any grade, 0%), anemia (abemaciclib: any grade, 46.0%; grade ≥ 3, 9.5%; placebo: any grade, 3.2%; grade ≥ 3, 3.2%), and thrombocytopenia (abemaciclib: any grade, 33.3%; grade ≥ 3, 4.8%; placebo: any grade, 0%). TEAEs of elevated alanine aminotransferase (ALT; abemaciclib: any grade, 34.9%; grade ≥ 3, 9.5%; placebo: any grade, 3.2%; grade ≥ 3, 0%) and aspartate aminotransferase (AST; abemaciclib: any grade, 30.2%; grade ≥ 3, 6.3%; placebo: any grade, 6.5%; grade ≥ 3, 0%) were also more common in the abemaciclib group compared with placebo.

**Table 3:** Treatment-emergent adverse events occurring in ≥ 20% of Japanese patients by grade

≥ 20% in either group, *n* (%)	Abemaciclib + fulvestrant (*N* = 63)			Placebo + fulvestrant (*N* = 31)		
	All	Grade 3	Grade 4	All	Grade 3	Grade 4
Any	63 (100)	43 (68.3)	4 (6.3)	31 (100)	7 (22.6)	0 (0.0)
Diarrhea	60 (95.2)	9 (14.3)	0 (0.0)	8 (25.8)	1 (3.2)	0 (0.0)
Neutropenia	50 (79.4)	27 (42.9)	1 (1.6)	0 (0.0)	0 (0.0)	0 (0.0)
Leukopenia	42 (66.7)	13 (20.6)	0 (0.0)	0 (0.0)	0 (0.0)	0 (0.0)
Anemia	29 (46.0)	6 (9.5)	0 (0.0)	1 (3.2)	1 (3.2)	0 (0.0)
Nausea	23 (36.5)	3 (4.8)	0 (0.0)	7 (22.6)	1 (3.2)	0 (0.0)
ALT increased	22 (34.9)	5 (7.9)	1 (1.6)	1 (3.2)	0 (0.0)	0 (0.0)
Thrombocytopenia	21 (33.3)	2 (3.2)	1 (1.6)	0 (0.0)	0 (0.0)	0 (0.0)
Abdominal pain	20 (31.7)	0 (0.0)	0 (0.0)	5 (16.1)	0 (0.0)	0 (0.0)
AST increased	19 (30.2)	4 (6.3)	0 (0.0)	2 (6.5)	0 (0.0)	0 (0.0)
Dysgeusia	18 (28.6)	0 (0.0)	0 (0.0)	1 (3.2)	0 (0.0)	0 (0.0)
Stomatitis	18 (28.6)	1 (1.6)	0 (0.0)	7 (22.6)	0 (0.0)	0 (0.0)
Vomiting	17 (27.0)	1 (1.6)	0 (0.0)	3 (9.7)	0 (0.0)	0 (0.0)
Blood creatinine increased	15 (23.8)	0 (0.0)	0 (0.0)	0 (0.0)	0 (0.0)	0 (0.0)
Decreased appetite	15 (23.8)	2 (3.2)	0 (0.0)	5 (16.1)	0 (0.0)	0 (0.0)
Pyrexia	15 (23.8)	0 (0.0)	0 (0.0)	3 (9.7)	0 (0.0)	0 (0.0)
Rash	15 (23.8)	0 (0.0)	0 (0.0)	3 (9.7)	0 (0.0)	0 (0.0)
Headache	14 (22.2)	1 (1.6)	0 (0.0)	8 (25.8)	0 (0.0)	0 (0.0)
Nasopharyngitis	10 (15.9)	0 (0.0)	0 (0.0)	10 (32.3)	1 (3.2)	0 (0.0)

MedDRA version 19.1; CTCAE version 4. Data cutoff date: February 14, 2017

*ALT*, alanine aminotransferase, *AST* aspartate aminotransferase, *CTCAE* Common Terminology Criteria for Adverse Events, *MedDRA* Medical Dictionary for Regulatory Activities, *N* number of patients in population, *n* number of patients

Additional TEAEs to note include fatigue, which was reported less frequently in the Japanese subpopulation (abemaciclib: any grade, 11.1%; grade ≥ 3, 1.6%; placebo: any grade, 16.1%; grade ≥ 3, 0%) compared with the overall safety population (abemaciclib: any grade, 39.9%; grade ≥ 3, 2.7%; placebo: any grade, 26.9%; grade ≥ 3, 0.4%; [13]). In addition, one patient in the abemaciclib group in the Japanese safety population had 1 event of grade 1 pneumonitis (interstitial lung disease; ILD).

The AEs leading to abemaciclib dose adjustments were most commonly diarrhea (dose reduction: 23.8%; dose omission: 22.2%) and neutropenia (dose reduction: 12.7%; dose omission: 33.3%) (Online Resource 3), in accordance with previous findings in the overall population (dose reductions: diarrhea, 18.8%; neutropenia, 10.0%; dose omissions: diarrhea, 18.8%; neutropenia, 16.3%) [13]. Four (6.3%) patients in the abemaciclib group discontinued study treatment due to an AE (placebo, 0%), which included 2 events of drug-induced liver injury and 1 event each of ALT and AST elevation.

### Quality of life

**Fig. 3:**
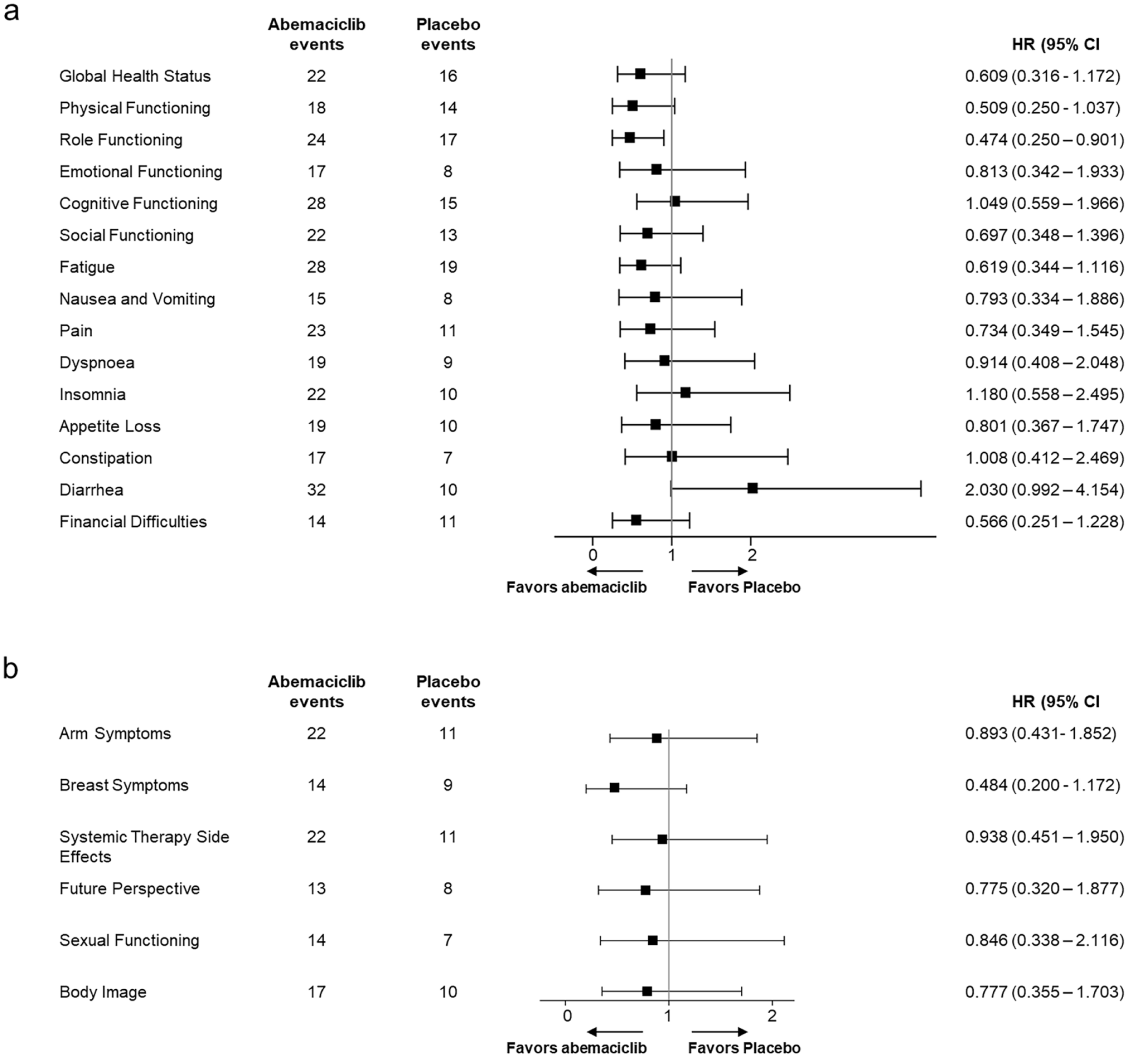
Forest plots of time to sustained deterioration of individual scales on the EORTC QLQ-C30 and QLQ-BR23.** a** EORTC QLQ-C30; and **b** EORTC QLQ-BR23 assessments in the Japanese subpopulation of MONARCH 2. In (**a**), deterioration of symptoms represents an increase in scores of ≥ 10; deterioration of global health status and functioning scores represents a decrease in scores of ≥ 10. In (**b**), deterioration of symptoms of Body Image, Sexual Functioning, and Future Perspectives represents a decrease in scores of ≥ 10; deterioration of symptoms of Systemic Therapy side effects, Arm, and Breast represents an increase in score of ≥ 10. Death was included as a deterioration event and follow-up improvement was taken into consideration. Data cutoff date: February 14, 2017. *CI* confidence interval, *EORTC* European Organization for Research and Treatment of Cancer, *HR* hazard ratio, *QLQ-BR23* Quality of Life Questionnaire-Breast subscale, 23 items, *QLQ-C30*, Quality of Life Questionnaire-Core 30

At baseline, the EORTC QLQ-C30 global health status score and the EORTC QLQ-C30 and QLQ-BR23 functional and symptom scores were generally similar between treatment groups (Table 4). Change from baseline for assessment items on the EORTC QLQ-C30 and QLQ-BR23 were not substantially different between treatment groups, except for a numerically lower diarrhea score in the placebo group which met the clinically meaningful threshold (mean [SE]: abemaciclib, 28.2 [2.1]; placebo, 2.6 [2.9]). There were no TTSD differences between treatment arms (confidence intervals cross 1) for all items except role functioning, which favored the abemaciclib group (Fig. 3a and b). The other notable exception was the diarrhea item, which numerically favored the placebo group.

**Table 4:** Mean baseline scores and within-treatment group change from baseline: EORTC QLQ-C30 and QLQ-BR23

Assessment	Baseline score Mean (SD)		Change from baseline^a^ Least squares mean (SE)	
	Abemaciclib + fulvestrant (*n* = 62)	Placebo + fulvestrant (*n* = 31)	Abemaciclib + fulvestrant (*n* = 62)	Placebo + fulvestrant (*n* = 31)
EORTC QLQ-C30^b^				
Global health status	70.0 (20.3)	67.7 (23.6)	− 5.4 (1.8)	− 5.6 (2.5)
Functional scales				
Physical	82.8 (19.1)	84.3 (19.4)	− 0.20 (1.3)	− 3.1 (1.8)
Role	83.3 (23.0)	85.0 (25.9)	− 2.8 (1.8)	− 7.9 (2.5)
Emotional	76.4 (20.0)	79.8 (18.6)	6.1 (1.2)	4.9 (1.7)
Cognitive	83.6 (18.2)	87.1 (15.3)	− 1.9 (1.6)	− 3.4 (2.2)
Social	85.5 (23.5)	85.0 (21.7)	0.4 (1.6)	0.7 (2.2)
Symptom scales				
Fatigue	25.1 (19.0)	26.5 (21.8)	5.1 (1.8)	6.6 (2.5)
Nausea and vomiting	2.2 (9.3)	2.2 (5.7)	2.5 (0.8)	1.6 (1.1)
Pain	23.1 (24.0)	25.3 (25.8)	− 2.8 (1.7)	4.1 (2.4)
Dyspnea	12.4 (17.3)	16.1 (24.1)	4.8 (1.7)	− 0.3 (2.4)
Insomnia	14.0 (18.6)	22.6 (30.3)	0.6 (2.1)	3.9 (2.9)
Appetite loss	10.2 (18.7)	11.8 (20.3)	3.8 (1.7)	1.1 (2.3)
Constipation	8.6 (18.0)	14.0 (18.8)	3.4 (1.8)	0.9 (2.5)
Diarrhea	6.5 (13.3)	4.3 (11.4)	28.2 (2.1)	2.6 (2.9)
Financial difficulties	14.0 (26.0)	6.5 (15.9)	− 2.6 (1.2)	− 0.5 (1.7)
EORTC QLQ-BR23^b^ Functional scales				
Body image	71.4 (22.9)	79.6 (21.7)	0.9 (1.8)	− 1.2 (2.5)
Sexual functioning	3.5 (9.6)	6.5 (12.7)	− 0.8 (0.7)	0.1 (1.0)
Future perspectives	42.5 (29.1)	43.0 (28.8)	15.9 (2.3)	17.6 (3.2)
Symptom scales				
Systemic therapy side effects	13.7 (11.2)	14.6 (10.6)	5.8 (0.9)	2.1 (1.3)
Breast	16.9 (20.1)	20.4 (21.7)	− 4.7 (1.3)	− 1.7 (1.8)
Arm	15.9 (16.8)	17.6 (19.4)	− 0.8 (1.5)	1.7 (2.1)

Data cutoff date: February 14, 2017

*EORTC* European Organization for Research and Treatment of Cancer, *MMRM* mixed model-repeated measures, *n* number of subjects in the population with baseline and post-baseline value for the question at the specified visit, *QLQ-BR23* Quality of Life Questionnaire-Breast subscale, 23 items, *QLQ-C30* Quality of Life Questionnaire-Core 30, *SD* standard deviation, *SE* standard error

^a^Change from baseline was assessed with a Type 3 sums of squares MMRM model (Change from Baseline = Treatment + Visit + Treatment*Visit + Baseline), including all cycles for which at least 25% of patients in each group have an assessment for each of the functional and symptom scales. Unstructured covariance structure was used for the MMRM model

^b^Deterioration of symptoms is represented by an increase in scores; deterioration of global health status and functioning scores is represented by a decrease in scores

### Overall survival, updated PFS, and updated safety

The cutoff date for the OS analysis was June 20, 2019, with a median follow-up time of 47.7 months. The updated PFS analysis at this data cutoff was consistent with the final PFS analysis (Online Resource 4a; HR: 0.562; 95% CI: 0.338–0.934). The updated median PFS was 23.8 months in the abemaciclib group compared with 14.3 months in the placebo group, reflecting a 9.5-month improvement in PFS with abemaciclib. The 36-month PFS rate was 38.6% (95% CI: 26.4–50.6) in the abemaciclib group and 11.1% (95% CI: 2.9–25.4) in the placebo group (treatment effect difference 27.5% [95% CI: 10.6–44.4]). Median OS (Online Resource 4b) was not reached in the abemaciclib group of the Japanese subpopulation whereas OS in the placebo group was 47.3 months (HR: 0.755; 95% CI: 0.390–1.463).

A higher proportion of patients in the placebo group (*n* = 29 of 31; 93.5%) received post-discontinuation chemotherapy compared with the abemaciclib group (*n* = 44 of 64; 68.8%), including > fivefold higher usage of CDK4 and CDK6 inhibitors as post-discontinuation therapy (placebo, 41.9%; abemaciclib, 7.8%). Post-discontinuation CDK4 and CDK6 inhibitor usage was lower in the overall ITT population (placebo, 17.0%; abemaciclib, 5.8%) [12]. The time to first post-discontinuation chemotherapy showed a trend for improvement in the abemaciclib group compared with the placebo control, with median TTC 52.3 versus 26.8 months, respectively (HR: 0.651; 95% CI: 0.353–1.198) and median CFS 50.2 versus 26.8 months, respectively (HR: 0.609; 95% CI: 0.344–1.076; Online Resource 5). A summary of the types of first-line post-discontinuation therapies is included in Online Resource 6. Of the 73 patients in the Japanese subpopulation who received any post-discontinuation therapy, the first subsequent therapy was chemotherapy for 32 patients (43.8%), single-agent ET for 28 patients (38.4%), and everolimus-based therapy for 6 patients (8.2%). In comparison, of the 461 patients in the overall ITT population of MONARCH 2 who received post-discontinuation therapy, 209 (45.3%), 119 (25.8%), and 80 (17.4%) received chemotherapy, single-agent ET, and everolimus-based therapy, respectively, as the first subsequent therapy [12].

The updated safety analysis indicated similar results to the primary analysis (Online Resource 7), with the proportions of grade ≥ 3 TEAEs little changed from the primary endpoint analysis (abemaciclib: grade 3, 66.7%; grade 4, 7.9%; placebo: grade 3, 22.6%; grade 4, 6.5%). Diarrhea, neutropenia, and leukopenia were again the most common TEAEs and were reported at a higher frequency in the abemaciclib group compared with the placebo group (abemaciclib: diarrhea: any grade, 95.2%, [grade ≥ 3 14.3%]; placebo, any grade, 35.5%; [grade ≥ 3, 3.2%]; abemaciclib: neutropenia: any grade, 81.0% [grade ≥ 3, 52.4%]; placebo, any grade, 0%]; leukopenia: abemaciclib, any grade, 69.8% [grade ≥ 3, 23.8%]; placebo, any grade, 0%). The incidence of ILD reported in the Japanese safety population treated with abemaciclib plus fulvestrant was 4.8% (*n* = 3), including 1 (1.6%) event of grade 1 and 2 events (3.2%) of grade 2 pneumonitis.

## Discussion

To gain a better understanding of the efficacy and safety of abemaciclib in Japanese breast cancer patients, the current analysis examined the Japanese subpopulation of MONARCH 2, a phase 3 study of abemaciclib plus fulvestrant in a global population of patients with HR+ , HER2− ABC who had progressed on prior ET [12, 13, 23]. Collectively, our results indicate that in the setting of advanced, ET-resistant, HR+ , HER2− breast cancer, Japanese patients derived benefit from the addition of abemaciclib to fulvestrant, with outcomes broadly consistent with those of the overall ITT population [12, 13]. At the time of the final PFS analysis, the addition of abemaciclib to fulvestrant resulted in improvement in median PFS by 6.9 months in the Japanese subpopulation (HR: 0.672; 95% CI: 0.380–1.189; abemaciclib, 21.2 months versus placebo, 14.3 months) whereas abemaciclib resulted in a 7.1-month improvement in median PFS in the overall ITT population (HR: 0.553; 95% CI: 0.449–0.681; abemaciclib, 16.4 months versus placebo, 9.3 months) [13]. At the time of the OS analysis, which occurred 27 months following the final PFS analysis, an updated assessment showed a 9.5-month improvement in median PFS with abemaciclib in the Japanese subpopulation (HR: 0.562; 95% CI: 0.338–0.934; overall population: HR: 0.536; 95% CI: 0.445–0.645 [12]) and a 27.5% treatment effect difference in PFS rates at 36 months. The OS HR for the Japanese subpopulation (HR: 0.755; 95% CI: 0.390–1.463) was favorable and consistent with that observed in the overall ITT population (HR: 0.757; 95% CI: 0.606–0.945) [12]. Median OS was not reached in the abemaciclib group of the Japanese subpopulation (whereas median OS was improved in the abemaciclib group by 9.4 months compared with placebo in the overall MONARCH 2 ITT population [12]), indicating that further follow-up is needed in the Japanese subpopulation. Nevertheless, this result is informative to understanding the effect of abemaciclib plus fulvestrant treatment in Japanese patients. TTC and CFS in the Japanese subpopulation also showed trends consistent with TTC and CFS in the overall population, indicating in both analysis populations that abemaciclib delayed the need for subsequent chemotherapy, an important outcome for patients with incurable disease, and that the initial effect of abemaciclib persists beyond the initial progression.

The PK and safety profiles of the Japanese subpopulation were also similar to those of the overall population. In the Japanese subpopulation, plasma concentrations of abemaciclib were as expected based on prior studies [33]. TEAEs were largely manageable with dose adjustments and/or supportive therapy, and few discontinuations occurred due to TEAEs. As in the global population, diarrhea and hematologic events were the most common TEAEs in the abemaciclib group. However, some TEAEs occurred at a higher frequency in the Japanese subpopulation compared with the overall population [13], including a higher incidence of grade 2 and 3 (but not grade 4) neutropenia and leukopenia, Similar to this finding, a higher frequency of hematological toxicities was previously observed in the Japanese subpopulation of the global phase 3 study of another CDK4 and CDK6 inhibitor, palbociclib, in women with HR + , HER2- ABC, with a higher incidence of ≥ grade 3 neutropenia in Japanese patients treated with palbociclib compared with the overall study population [9]. Although the pathogenesis of this finding is unknown, this may indicate a drug-class effect. Despite the higher incidence of neutropenia in the Japanese MONARCH 2 subpopulation, neutropenia was not associated with an increase in severe infection or febrile neutropenia, and there were no discontinuations due to neutropenia in the Japanese subpopulation.

This study revealed a higher incidence of increased ALT and AST, predominantly low grade, in abemaciclib-treated patients in the Japanese subpopulation. This result is in accordance with previous findings for the CDK4 and CDK6 inhibitor, ribociclib, which had higher liver toxicity in Japanese patients [34]. In the current analysis, no cases of Hy’s law were observed, but one event each of elevated ALT and elevated AST led to study discontinuation, underscoring the importance of regular blood monitoring during abemaciclib treatment.

ILD is a well-recognized, potentially serious complication of many different cancer agents [35, 36] and a class side effect of CDK4 and CDK 6 inhibitors [37]. In MONARCH 2, a slightly higher incidence of ILD was found in the Japanese safety population (3 events; 4.8%) compared with the overall safety population (12 events; 2.7%), indicating the need for regular monitoring of patients treated with abemaciclib for symptoms of ILD.

Global HRQoL, most symptoms, and functioning scales did not meet the threshold for clinically meaningful differences for the treatment arms. The only exception to this was the QLQ-C30 diarrhea score, which favored the placebo arm with clinically meaningful differences. There were no TTSD differences between treatment arms for global HRQoL, most symptoms (except diarrhea), or functioning (except role function). These findings are in accordance with a higher proportion of patients treated with abemaciclib reporting TEAEs of diarrhea compared with the placebo group. However, diarrhea appeared to be effectively managed by dose adjustments and supportive care, as no patients discontinued due to diarrhea in the Japanese subpopulation. Collectively, the current results demonstrate that patients treated with abemaciclib plus fulvestrant did not experience a clinically meaningful detriment in their HRQoL in terms of general health status and across multiple functional and symptom scales and are in agreement with the HRQoL findings in the overall MONARCH 2 study population [23].

These results should be considered in light of the limitations of this analysis. Notably, the sample size in the Japanese subpopulation is small, and statistical hypothesis testing was not applied to this analysis. Additional considerations include the noted differences between the Japanese subpopulation and the overall population that potentially could affect response to treatment in terms of both efficacy outcomes and tolerability, e.g., the Japanese subpopulation had a higher proportion of pre/perimenopausal women and a lower proportion of abemaciclib-treated patients with primary ET resistance and prior chemotherapy compared to the overall population. In addition, the Japanese subpopulation had higher usage of post-discontinuation CDK4 and CDK 6 inhibitors.

## Conclusion

Consistent with the findings of the global study [12, 13, 23], Japanese patients in the MONARCH 2 study derived benefit from the addition of abemaciclib to fulvestrant in terms of improved PFS and delayed need for subsequent chemotherapy. In the Japanese subpopulation, abemaciclib plus fulvestrant had a manageable safety profile, without clinically meaningful differences from placebo plus fulvestrant across most HRQoL dimensions evaluated.

## Supplementary Information

Below is the link to the electronic supplementary material.

Supplementary file1 (DOCX 361 KB)
