# Supplementary material for: Japanese subpopulation analysis of MONARCH 2: phase 3 study of abemaciclib plus fulvestrant for treatment of hormone receptor-positive, human epidermal growth factor receptor 2-negative breast cancer that progressed on endocrine therapy
Source: Breast Cancer. 2021 Apr 1;28(5):1038–50. doi: 10.1007/s12282-021-01239-8 (PMC8354907; doi:10.1007/s12282-021-01239-8)
Supplement: Supplementary file 1 — Supplementary file1 (DOCX 361 KB) [file 12282_2021_1239_MOESM1_ESM.docx]

**Online resources for:**

*Breast Cancer*

Japanese subpopulation analysis of MONARCH 2: phase 3 study of abemaciclib plus fulvestrant for treatment of hormone receptor-positive, human epidermal growth factor receptor 2-negative breast cancer that progressed on endocrine therapy

Kenichi Inoue, Norikazu Masuda, Hiroji Iwata, Masato Takahashi, Yoshinori Ito, Yasuo Miyoshi, Takahiro Nakayama, Hirofumi Mukai, Jan-Stefan van der Walt, Joji Mori, Sachi Sakaguchi, Tsutomu Kawaguchi, Yoshinori Tanizawa, Antonio Llombart-Cussac, George W. Sledge, Jr, Masakazu Toi

**Corresponding author:** Masakazu Toi, Email: [toi@kuhp.kyoto-u.ac.jp](mailto:toi@kuhp.kyoto-u.ac.jp);


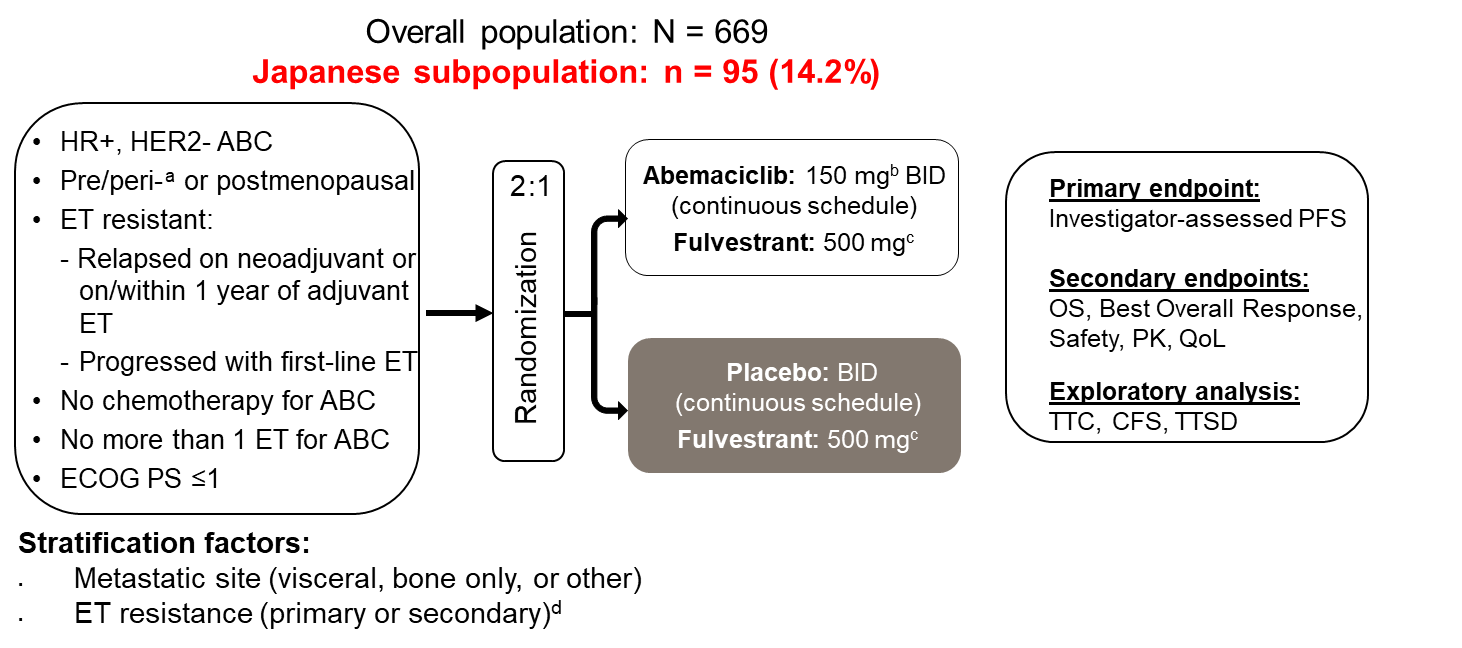


**Online Resource 1 MONARCH 2 study design** MONARCH 2 was a global, randomized, double-blind, placebo-controlled phase 3 study in women with HR+, HER2- ABC who progressed during neoadjuvant or adjuvant ET within 1 year after adjuvant ET or while receiving first-line ET for ABC. Patients were randomized 2:1 to receive abemaciclib plus fulvestrant or placebo plus fulvestrant. The primary endpoint was PFS, and key secondary endpoints included OS, best overall response, safety, PK, and QoL outcomes. To determine progression and response, tumors were measured by computed tomography or magnetic resonance imaging within 28 days before random assignment (baseline) and then every 8 weeks the first year, every 12 weeks thereafter, and within 2 weeks of clinical progression. Treatment-emergent adverse events were recorded and graded based on National Cancer Institute Terminology criteria, version 4, from baseline to follow-up. Interim and final PFS analyses were preplanned to occur at approximately 265 and 378 investigator-assessed PFS events, respectively, in the ET-pretreated patients of the overall ITT population, for 90% power with a one-sided alpha of 0.025, assuming a true hazard ratio (HR) of 0.703. Interim and final OS analyses were preplanned to occur when approximately 331 and 441 OS events, respectively, had occurred in the ET-pretreated patients in the overall ITT population. A gate-keeping strategy between PFS and OS was used, with OS to be tested only if the test of PFS was significant.

^a^Required to receive gonadotropin-releasing hormone agonist.

^b^Dose reduced by protocol amendment in all new and ongoing patients from 200 mg to 150 mg BID after 178 patients enrolled.

^c^Fulvestrant administered per label.

^d^Primary clinical ET resistance per ESMO guidelines: 1) In the adjuvant setting, recurrence within the first 2 years of adjuvant ET while on ET; 2) in the advanced/metastatic setting, progression within the first 6 months of initiating first-line ET while on ET. Patients receiving prior ET who did not meet the definition of primary resistance were considered to have secondary clinical resistance.[24, 25]

ABC, advanced breast cancer; BID, twice daily dose; CFS, chemotherapy-free survival; ECOG PS, Eastern Cooperative Oncology Group Performance Status; ET, endocrine therapy; HER2-, human epidermal growth factor receptor 2 negative; HR+, hormone receptor-positive; N, number of patients in analysis population; n, number of patients in category or group; OS, overall survival; PFS, progression-free survival; PK, pharmacokinetics; QoL, quality of life; TTC, time to post-discontinuation chemotherapy; TTSD, time to sustained deterioration.

**
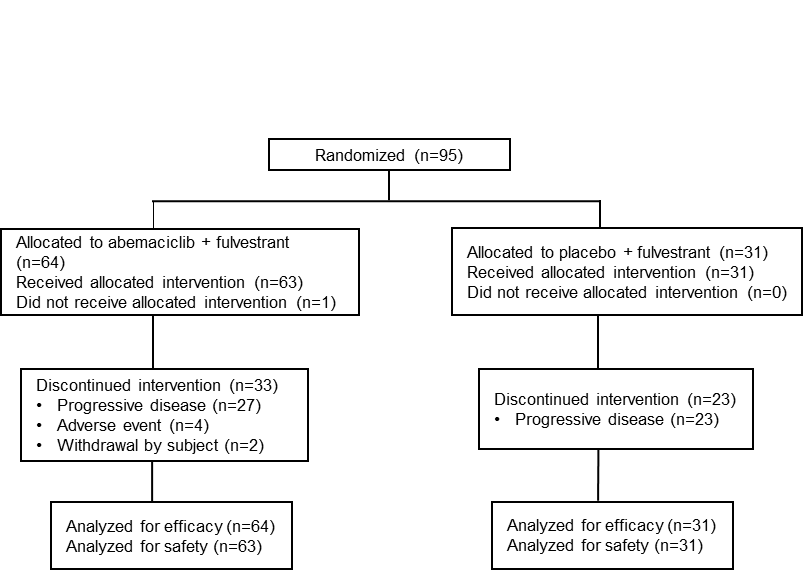
**

**Online Resource 2 Patient disposition** Data cut-off date: February 14, 2017.

**Online Resource 3 Dose adjustment and exposure summary in the MONARCH 2 Japan subpopulation**

|  | **Abemaciclib + Fulvestrant (n=63)** | **Placebo + Fulvestrant (n=31))** |
| --- | --- | --- |
| **Duration of therapy (abemaciclib), weeks** |  |  |
| Mean (SD) | 57.7 (38.9) | 59.2 (30.3) |
| Median | 64.7 | 65.0 |
| Minimum – maximum | 2.1 – 114.0 | 6.3 – 106.0 |
| **Duration of therapy (fulvestrant), weeks** |  |  |
| Mean (SD) | 62.6 (36.5) | 60.0 (29.6) |
| Median | 75.0 | 65.0 |
| Minimum – maximum | 2.0 – 114.0 | 8.0 – 106.0 |
| **Dose intensity**^a^ **(abemaciclib), mg per day** |  |  |
| Mean (SD) | 223.0 (67.8) | 302.9 (25.9) |
| Median | 231.2 | 298.3 |
| Minimum – maximum | 76.6 – 310.5 | 270.6 – 390.8 |
| **Relative dose intensity**^a^ **(abemaciclib), %** |  |  |
| Mean (SD) | 68.1 (22.1) | 92.2 (9.7) |
| Median | 69.7 | 95.0 |
| Minimum – maximum | 25.5 – 103.5 | 73.5 – 112.5 |
| **Dose reductions due to AEs (abemaciclib), n (%)** | 34 (54.0) | 1 (3.2) |
| Reasons, ≥10% in either group |  |  |
| Diarrhea | 15 (23.8) | 0 (0.0) |
| Neutropenia | 8 (12.7) | 0 (0.0) |
| **Dose omissions due to AEs (abemaciclib), n (%)** | 52 (82.5) | 6 (19.4) |
| Reasons, ≥10% in either group |  |  |
| Diarrhea | 14 (22.2) | 0 (0.0) |
| Neutropenia | 21 (33.3) | 0 (0.0) |
| Anemia | 7 (11.1) | 0 (0.0) |

Data cut-off date February 14, 2017.

^a^Dose intensity refers to the actual total amount of drug administered per day.

^b^Relative dose intensity refers to the percentage of actual amount of drug taken relative to amount of drug prescribed.

AEs, adverse events; n, number of patients in category or group; SD, standard deviation.


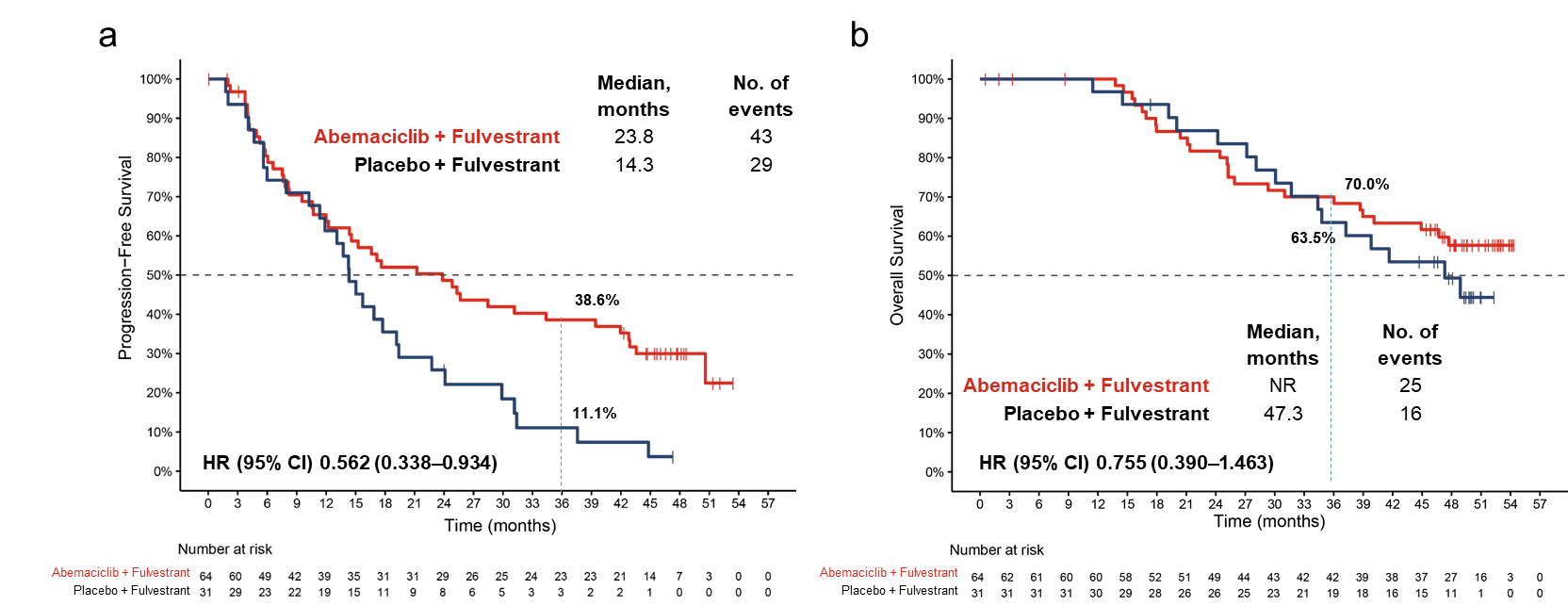


**Online Resource 4 Updated progression-free survival and** **overall survival a**. Updated PFS; and **b**. OS at the data cut-off date of June 20, 2019, for the MONARCH 2 Japanese subpopulation. PFS was defined as the time from the date of randomization until the date of radiographic documentation of progression, based on investigator assessment, or the date of death, whichever was earlier. OS was defined as the time from the date of randomization to the date of death from any cause. The curves and medians (95% CI) were estimated using the Kaplan-Meier method. CI, confidence interval; HR, hazard ratio; No., number; OS, overall survival; PFS, progression-free survival.


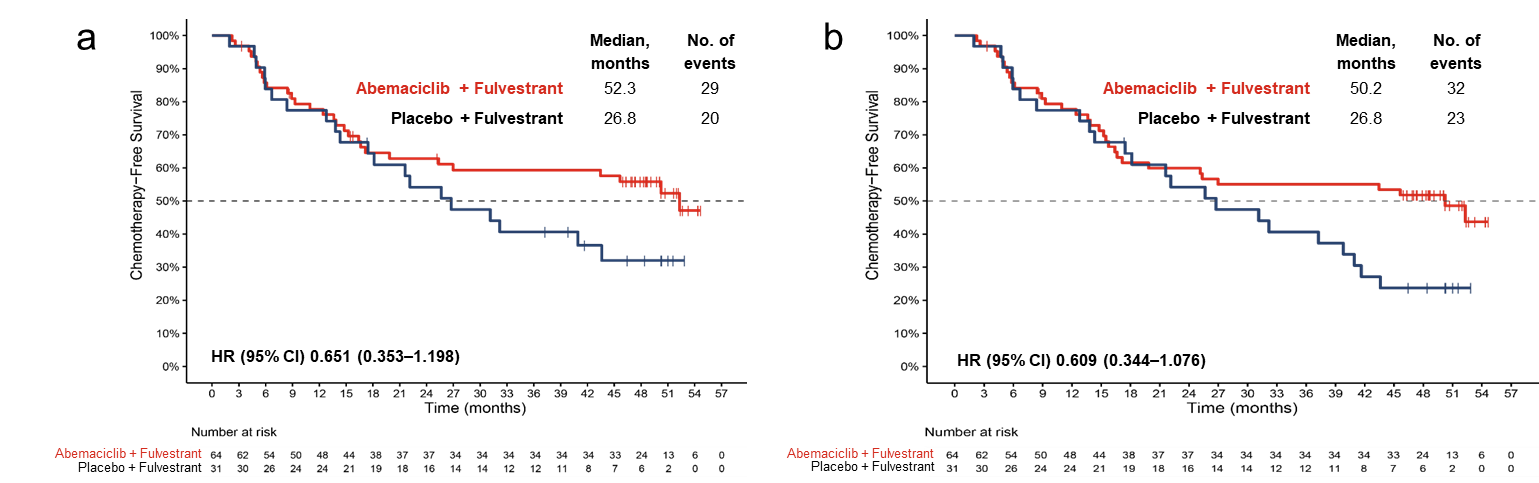


**Online Resource 5 Time to first post-discontinuation chemotherapy** Exploratory endpoints at the data cut-off date of June 20, 2019, for the MONARCH 2 Japanese subpopulation. **a**. TTC; **b**. CFS. Both TTC and CFS show durations from the time from randomization to initiation of first post-discontinuation chemotherapy or death, but the TTC graph shows data with censoring of patients who died prior to initiation of post-discontinuation chemotherapy. The curves and medians (95% CI) were estimated using the Kaplan-Meier method. CFS, chemotherapy-free survival; CI, confidence interval; HR, hazard ratio; TTC, time to chemotherapy

**Online Resource 6 First subsequent line of post-discontinuation therapy**

|  | **Japanese ITT Population**  **(N=95)** | | **Overall ITT Population**  **(N=669)** | |
| --- | --- | --- | --- | --- |
| **First subsequent line of PDT** | **Abemaciclib + Fulvestrant (n=64)^a^** | **Placebo + Fulvestrant (n=31)** | **Abemaciclib + Fulvestrant (n=446)** | **Placebo + Fulvestrant (n=223)** |
| Number of patients receiving any PDT | 44 | 29 | 281 | 180 |
| Chemotherapy, n  Median duration, months (95% CI) | 21  3.72 (1.61, 4.41) | 11  3.95 (2.30, 13.38) | 130  4.01 (4.41, 5.56) | 79  4.60 (2.76, 5.65) |
| Endocrine only, n  Median duration, months (95% CI) | 16  9.44 (27.9, 31.10) | 12  4.41 (1.81, 7.76) | 72  5.29 (3.72, 9.47) | 47  4.83 (3.78, 6.48) |
| Everolimus-based therapy, n  Median duration, months (95% CI) | 3  2.20 (0.03, 2.50) | 3  16.31 (8.52, 17.69) | 48  4.50 (3.02, 6.64) | 32  8.79 (5.69, 14.50) |
| Other^b^, n  Median duration, months (95% CI) | 4  3.22 (2.30, NR) | 3  NR (2.10, NR) | 31  8.02 (3.25, 20.12) | 22  14.76 (5.06, 19.13) |
| Not applicable^c^, n | 20 | 2 | 165 | 43 |

Data cut-off date: June 20, 2019.

^a^One patient with no post-baseline tumor assessment discontinued treatment (withdrawal by subject) and received PDT.

^b^Includes other CDK4/CDK6 inhibitors and non-CDK4/CDK6 inhibitors.

^c^Includes patients on-treatment without PFS event and patients who have experienced a PFS event but did not receive PDT.

CDK4 and CDK6, cyclin dependent kinase 4 and 6; CI, confidence interval; ITT, intent-to-treat; N, patients in population; n, patients in category or group; NR, not reached; PDT, post-discontinuation therapy; PFS, progression-free survival.

**Online Resource 7 Updated safety: Treatment-emergent adverse events occurring in ≥20% of Japanese patients by grade**

|  | **Abemaciclib + Fulvestrant (N=63)** | | | **Placebo + Fulvestrant (N=31)** | | |
| --- | --- | --- | --- | --- | --- | --- |
| **≥20% in either group, n (%)** | **All** | **Grade 3** | **Grade 4** | **All** | **Grade 3** | **Grade 4** |
| Any | 63 (100) | 42 (66.7) | 5 (7.9) | 31 (100) | 7 (22.6) | 2 (6.5) |
| Diarrhea | 60 (95.2) | 9 (14.3) | 0 (0.0) | 11 (35.5) | 1 (3.2) | 0 (0.0) |
| Neutropenia | 51 (81.0) | 32 (50.8) | 1 (1.6) | 0 (0.0) | 0 (0.0) | 0 (0.0) |
| Leukopenia | 44 (69.8) | 15 (23.8) | 0 (0.0) | 0 (0.0) | 0 (0.0) | 0 (0.0) |
| Anemia | 31 (49.2) | 6 (9.5) | 0 (0.0) | 3 (9.7) | 2 (6.5) | 0 (0.0) |
| ALT increased | 27 (42.9) | 6 (9.5) | 1 (1.6) | 1 (3.2) | 0 (0.0) | 0 (0.0) |
| Nausea | 26 (41.3) | 3 (4.8) | 0 (0.0) | 7 (22.6) | 1 (3.2) | 0 (0.0) |
| AST increased | 25 (39.7) | 5 (7.9) | 0 (0.0) | 2 (6.5) | 0 (0.0) | 0 (0.0) |
| Abdominal pain | 21 (33.3) | 0 (0.0) | 0 (0.0) | 5 (16.1) | 0 (0.0) | 0 (0.0) |
| Thrombocytopenia | 21 (33.3) | 2 (3.2) | 1 (1.6) | 0 (0.0) | 0 (0.0) | 0 (0.0) |
| Dysgeusia | 19 (30.2) | 0 (0.0) | 0 (0.0) | 1 (3.2) | 0 (0.0) | 0 (0.0) |
| Stomatitis | 19 (30.2) | 0 (0.0) | 1 (1.6) | 8 (25.8) | 0 (0.0) | 0 (0.0) |
| Vomiting | 19 (30.2) | 1 (1.6) | 0 (0.0) | 4 (12.9) | 0 (0.0) | 0 (0.0) |
| Blood creatinine increased | 17 (27.0) | 0 (0.0) | 0 (0.0) | 0 (0.0) | 0 (0.0) | 0 (0.0) |
| Pyrexia | 17 (27.0) | 0 (0.0) | 0 (0.0) | 5 (16.1) | 0 (0.0) | 0 (0.0) |
| Rash | 17 (27.0) | 0 (0.0) | 0 (0.0) | 3 (9.7) | 0 (0.0) | 0 (0.0) |
| Decreased appetite | 15 (23.8) | 2 (3.2)) | 0 (0.0) | 5 (16.1) | 0 (0.0) | 0 (0.0) |
| Headache | 15 (23.8) | 1 (1.6) | 0 (0.0) | 9 (29.0) | 0 (0.0) | 0 (0.0) |
| Upper respiratory tract infection | 15 (23.8) | 0 (0.0) | 0 (0.0) | 2 (6.5) | 0 (0.0) | 0 (0.0) |
| Injection site reaction | 14 (22.2) | 0 (0.0) | 0 (0.0) | 4 (12.9) | 0 (0.0) | 0 (0.0) |
| Nasopharyngitis | 11 (17.5) | 0 (0.0) | 0 (0.0) | 9 (29.0) | 1 (3.2) | 0 (0.0) |
| Constipation | 9 (14.3) | 0 (0.0) | 0 (0.0) | 8 (25.8) | 0 (0.0) | 0 (0.0) |

MedDRA version 22.0; CTCAE version 4; Data cut-off date: June 20, 2019.

ALT, alanine aminotransferase; AST, aspartate aminotransferase; CTCAE, Common Terminology Criteria for Adverse Events; MedDRA, Medical Dictionary for Regulatory Activities; N, number of patients in population; n, number of patients.
